# Adolescent males’ knowledge about safe sex in light of the Sustainable Development Goals

**DOI:** 10.1590/0034-7167-2024-0422

**Published:** 2025-06-20

**Authors:** Wallacy Jhon Silva Araújo, Danielle Laet Silva Galvão, Dayanne Caroline de Assis Silva, Gabriela Rodrigues Bragagnollo, Eliane Maria Ribeiro de Vasconcelos, Mariana Boulitreau Siqueira Campos Barros, Estela Maria Leite Meirelles Monteiro

**Affiliations:** I Universidade Federal de Pernambuco. Recife, Pernambuco, Brazil; II Universidade de São Paulo. Ribeirão Preto, São Paulo, Brazil

**Keywords:** Adolescent, Sexuality, Masculinity, Sustainable Development, Health Education, Adolescente, Sexualidad, Masculinidad, Desarrollo Sostenible, Educación en Salud

## Abstract

**Objectives:**

to understand adolescent males’ knowledge about safe sex in light of the Sustainable Development Goals of the 2030 Agenda.

**Methods:**

a qualitative study involving 18 male adolescents, aged 15 to 19 years, in a public school in Recife, Pernambuco, conducted between June and December 2019. A semi-structured instrument was used to conduct interviews, and thematic content analysis was based on the framework proposed by Bardin.

**Results:**

three thematic categories emerged: Adolescent Males’ Knowledge About Safe Sex; Weaknesses in Guidance on Safe Sex Within the Family Context; and School as a Privileged Space for Teaching and Learning About Safe Sex for Adolescent Males.

**Final Considerations:**

limitations in knowledge about safe sex have negative health repercussions, weakening family and school dialogues and compromising the understanding of risk factors that contribute to early and unprotected sexual initiation among adolescents and their partners.

## RESUMEN

**Objetivos:**

comprender el conocimiento de adolescentes masculinos sobre sexo seguro a la luz de los Objetivos de Desarrollo Sostenible de la Agenda 2030.

**Métodos:**

estudio cualitativo con 18 adolescentes masculinos, de entre 15 y 19 años, en una escuela pública de Recife/Pernambuco, realizado entre los meses de junio y diciembre de 2019. Se utilizo un instrumento semiestructurado para realizar las entrevistas y el análisis temático del contenido se fundamento en el marco teórico propuesto por Bardin.

**Resultados:**

surgieron tres categorias temáticas: Conocimiento de adolescentes masculinos sobre sexo seguro; Fragilidades en la orientación sobre sexo seguro en el contexto familiar; y La escuela como espacio privilegiado para la enseñanza-aprendizaje sobre sexo seguro para adolescentes masculinos.

**Consideraciones Finales:**

las limitaciones en el conocimiento sobre sexo seguro generan repercusiones perjudiciales para la salud, debilitando el diálogo familiar y escolar, y comprometiendo la comprensión de los factores de riesgo que contribuyen a la iniciación sexual temprana y desprotegida de adolescentes y sus parejas.

## INTRODUCTION

Adolescence is influenced by historical, social, and cultural conceptions linked to experiences of human sexuality^(1)^. During this period, adolescents engage in new behaviors, leading to their first romantic and sexual relationships, which allow them to seek the strengthening of their individual identities^(2)^. It is well-known that sexual practices extend beyond biological aspects and involve psychosocial and cultural characteristics shaped by social impositions, often molding male sexual behaviors that lack responsibility, ethical values, and morals, thus contributing to situations of vulnerability^(3)^.

Due to the presence of taboos, prejudices, and a lack of knowledge about safe sex during the exploration phase of sexual practices, inadequate information and ineffective communication with adolescents contribute to behaviors that undermine sexual self-care^(4)^.

With the advent of technological development, there has been an increase in the availability of sexual content online^(1)^. However, exposure to distorted information on this topic and the lack of safe virtual spaces for discussion contribute to the encouragement of early sexual initiation (ESI) without safety and responsibility, particularly among male adolescents^(3)^.

As a result of this deficit, unsafe sexual practices become recurrent, with primary consequences such as sexually transmitted infections (STIs), inconsistent contraceptive use, and unintended pregnancies^(5)^. These issues can lead to negative repercussions for adolescents, their families, and society, as well as increase healthcare costs across all levels of care^(1)^.

Therefore, in considering the guarantee of sexual and reproductive rights for adolescent males, goals have been established in line with the 2030 Agenda for Sustainable Development. These goals aim at the development of public policies and the implementation of actions directed at this age group to end the epidemics of AIDS and STIs; ensure universal access to sexual and reproductive health services, including family planning and information so that all boys and girls acquire the necessary knowledge and skills for their comprehensive development through quality education^(6)^.

In this context, the importance of the School Health Program and intersectoral and interdisciplinary educational health actions is highlighted, aiming to integrate and expand the school, family, and community context through a dialogic approach to this topic^(7)^.

## OBJECTIVES

To understand adolescent males’ knowledge about safe sex in light of the Sustainable Development Goals (SDG) of the 2030 Agenda.

## METHODS

### Ethical Aspects

The research project was approved by the Research Ethics Committee, in accordance with Resolution No. 466/12 of the National Health Council. Adolescents under eighteen years of age signed the Assent Form, while formal parental/guardian consent was required for their participation through the signing of the Parental/Guardian Informed Consent Form prior to data collection. Anonymity and confidentiality of personal information were guaranteed by assigning codenames to the participants.

### Theoretical-Methodological Framework

The theoretical-methodological framework used was the 2030 Agenda for the SDGs. This choice facilitated an analysis focused on achieving objectives that promote interconnected actions for health and well-being, quality education, and gender equality^(6)^.

### Type of Study

This was a descriptive study with a qualitative approach, guided by the Consolidated Criteria for Reporting Qualitative Research (COREQ)^(8)^.

### Methodological Procedures

The study population consisted of self-declared male adolescents, aged between 15 and 19 years, who had already initiated sexual practices. This age range was selected to allow for a detailed exploration of the topic with students in the maturation phase. Adolescents with special educational needs, as diagnosed by the medical team, and students who were already participating in other studies on the topic were excluded.

The recruitment of participants employed snowball sampling^(9)^, wherein the first participant was recommended by the school’s pedagogical coordination due to their role as a school leader, initiating the network of new participants. The delimitation of participants followed the criterion of theoretical saturation^(10)^, which was reached by the 14th interview when no new relevant information emerged for the study. Following the confirmation of saturation, four additional interviews were conducted to further validate the saturation process. The final sample consisted of 18 adolescents, and no dropouts occurred.

### Study Setting

The study was conducted in a public basic education school in the state of Pernambuco. The selected school offers basic education from the 5th grade of elementary school to the 3rd year of high school, but it does not have integrated actions with the School Health Program. The selection of the study setting was based on reports from the school administration and pedagogical team, which highlighted cases of alcohol and drug consumption, bullying, adolescent pregnancy, ESI, and STIs.

### Data Collection and Organization

The interviews were conducted between June and December 2019, preceded by the researcher’s preliminary integration into the school environment. An in-depth interview technique was used to capture objective data regarding the sociodemographic characterization of participants as well as a subjective question. The guiding question of the interview was previously validated by three specialists in the field of sexuality: a nurse, a pedagogue, and a psychologist.

A pilot test was conducted with interviews of three adolescents who were not included in the final sample. This process supported necessary adjustments to the presentation of the interview script. The semi-structured instrument for data collection was divided into two parts: the first part focused on gathering sociodemographic data, and the second part contained the key question for the qualitative interview^(11)^: “What knowledge do you consider necessary for male adolescents to feel prepared for safe sexual practices?”.

The interviews were conducted during morning and afternoon sessions, according to a schedule agreed upon with the pedagogical team to avoid interfering with curricular activities. A private setting was used, with an average interview duration of 40 minutes. The interviews were recorded using dual recording devices, transcribed, and subsequently validated by the adolescents through reading and approval of the transcriptions, scheduled via phone contact^(12)^.

### Data Analysis

Thematic content analysis, based on Bardin’s method, was used for data categorization and processing. This method involves three chronological phases: pre-analysis, which includes floating reading and initial organization of the research corpus; material exploration for coding and categorization; and treatment and interpretation of the results, supported by a reflective method^(13)^.

## RESULTS

Eighteen self-declared male adolescents participated, with a mean age of 18.3 years. Regarding sexual orientation, 13 adolescents identified as heterosexual, four as homosexual, and one as transgender; nine identified as Black, five as mixed-race, three as White, and one stated that they did not care about ethnic-racial self-identification. Concerning religious aspects, five were Evangelical, four Catholic, one practiced Candomblé, and eight reported having no religion, although they stated a belief in God.

Additionally, eight adolescents were in their third year, four in their second year, and four in their first year of high school, while two were in the ninth grade of elementary school. Most participants lived with their parents and siblings, and the majority had a household income of up to two minimum wages.

Based on participant narratives, three interrelated thematic categories emerged, titled: Adolescent Males’ Knowledge About Safe Sex; Weaknesses in Guidance on Safe Sex Within the Family Context; and School as a Privileged Space for Teaching and Learning About Safe Sex for Male Adolescents.

### Adolescent Males’ Knowledge About Safe Sex

Reports highlighted a lack of knowledge about safe sex practices, contributing to a situation of vulnerability due to the emergence of sexuality and the absence of risk perception related to adolescent health. There was a noted lack of access to knowledge that addresses the actual socio-emotional and cultural needs and specificities of this population group.

*I never received any information that effectively explained safe sex to me.* (Apolo)

*I knew very little, in fact, almost nothing. At that time, the only thing I knew was that for sex to happen, there had to be penetration.* (Himeneu)

*I was very clueless. I didn’t know anything. I just played video games. Only after my first sexual experience did I discover that ejaculating inside the girl’s vagina posed a risk of unplanned pregnancy.* (Anteros)

*I didn’t know anything. Actually, I think we don’t need any knowledge. There’s no need to look it up in books or online.* (Afrodite)

Some responses were limited to recommending condom use as a preventive method. However, participants still displayed weak knowledge about using condoms as a potential barrier for preventing contamination related to STIs. Adolescents’ fragile understanding of STIs limits their awareness of existing risks in unsafe sexual practices and weakens their adherence to condom use.

*Educationally, I had no knowledge; I didn’t have much information on what to do. The only information I had was about using condoms to prevent STIs, even though I didn’t know much about them.* (Zeus)

*I had no real sense of anything. I only knew what STIs were, like HIV, and what I should use at the time to prevent contamination.* (Éos)

*I had no knowledge. They just told me about using condoms to prevent STIs, like AIDS.* (Himeros)

Regarding emergency contraception, the morning-after pill was mistakenly associated with STI prevention as well as serving as a contraceptive method. Adolescents reported using this method to mitigate risky behaviors that occasionally occurred during unprotected sexual encounters, potentially leading to unplanned teenage pregnancies.

This fragile conception of the morning-after pill leaves male adolescents vulnerable to sexual and reproductive health risks, with social and emotional repercussions, including concerns about the risk of unplanned pregnancies and contracting STIs.

*If it happened that I had sex without a condom, the girl would have to take the morning-after pill the next day to prevent unplanned pregnancy and STI contamination. I knew that if I got a girl pregnant, it could cause problems for me.* (Hermes)

*We all know what happens during sex, and we can’t ever forget to take some medications the next day.* (Afrodite)

*My knowledge was about using condoms, and if it broke, we should buy the morning-after pill to prevent STI contamination and unplanned pregnancy.* (Ares)

### Weaknesses in Guidance on Safe Sex Within the Family Context

Although it is generally accepted that the family environment is a privileged emotional space for developing and acquiring knowledge about safe sex, most participants reported not discussing sexuality-related topics with their parents. This highlights the ongoing fragility of family relationships, associated with the taboos and prejudices that surround discussions on sexuality, making it difficult to understand safe sex as a healthy practice linked to human health issues. However, many parents and guardians are resistant to addressing the topic, as they believe it may encourage ESI. Additionally, it appears that adolescents feel more comfortable discussing safe sex with their mothers, due to the loving relationship they often maintain with their children, which is generally free from intolerance and prejudice.

*These topics were not discussed much; they were considered private for adults. My father didn’t like talking to me about these things, and I was always too shy to bring them up. Honestly, I preferred to search for information on the internet by myself.* (Zeus)

*My father and I never had much intimacy to discuss such topics. However, my mother always opened my eyes, emphasizing the importance of using a condom.* (Apolo)

*My parents never told me that if I ever had sexual relations, I should use a condom to avoid contracting an STI. We need to talk with our parents about these topics so that parents can advise their children and assess whether they are ready to start their sexual activities. Above all, they need to address topics like STI prevention.* (Deméter)

### School as a Privileged Space for Teaching and Learning About Safe Sex for Male Adolescents

Adolescents believe that school is a safe and suitable environment for acquiring information and developing reliable knowledge on sexuality issues; however, it was noted that access to this knowledge often occurs after the initiation of sexual activity. Additionally, among adolescents, there emerged a desire to express their opinions and thoughts democratically regarding condom use and STI prevention.

*Adolescents also have a voice and something to say. I believe that topics like this should be addressed in the school environment through discussion circles. I would like medical professionals or sexologists to address the topic of STIs in schools.* (Zeus)

*I never received information at home. I acquired it at school, two years after my sexual initiation. I learned about condoms and the necessary precautions to take when putting one on.* (Pothos)

*I had a lot of information. I learned at school through my teachers. They always taught me about the importance of using condoms to protect myself from theSTIs that exist in the world.* (Poseidon)

## DISCUSSION

The participants in this study were characterized as self-declared male adolescents with low socioeconomic status, mostly belonging to the Black population and lacking religious practice, which contributes to social vulnerability. The study demonstrated that this profile is strongly related to the socio-historical context that leads to fragile knowledge about safe sex and early sexual practices, culminating in situations of vulnerability regarding adolescent health^(14)^.

When addressing the topic of safe sex within the male context, it is necessary to consider individuals within the framework of multiple masculinities. To this end, it is important to recognize that the emergence of male identity is tied to the centrality of combining plurality and the hierarchy of masculinities, thereby deconstructing the concept of a single standard of power. Masculinities, in the plural, are socially constructed and vary across different sociocultural contexts over time, within potential spaces of identity, and throughout a man’s life^(15)^.

Thus, the representation of masculinity can be delineated in two ways: a traditional form, characterized by expressions of strength and virility, and a second form that encompasses manifestations of emotionality, sensitivity, creativity, and imagination—characteristics often denied to male identity in its holistic and plural dimension^(15)^.

It is essential to note that discussions on gender equality, as evidenced in SDG 5, have been approached within the scientific community, highlighting women’s rights in response to socially constructed machismo that contributes to situations of vulnerability. Therefore, it is crucial to emphasize the importance of male empowerment through self-awareness, aimed at establishing respectful relationships with shared responsibilities and fostering mutual support in building safe interpersonal and sexual relationships with partners^(7)^.

The vulnerabilities experienced by male adolescents in their social context are often shaped by traditional cultural expectations of hegemonic masculinity, underscoring the genuine need for investments that support the promotion of adequate and comprehensive knowledge about safe sex. This approach seeks to promote healthy sexual development, optimize comprehensive sexual education, and dismantle barriers to accessing sexual and reproductive health services^(16)^.

### Adolescent Males’ Knowledge About Safe Sex

The adolescents in this study demonstrated a fragile understanding of safe sex. A correlation was observed between the term “safe sex” and the use of male condoms as a protective method against STI transmission and unplanned pregnancies, aligning with findings from both national and international studies^(6, 17)^. However, it was evident that they did not consistently use protection during all sexual encounters, indicating that knowledge about contraceptive methods does not always translate into safe sexual practices^(17)^.

Furthermore, it was noted in this study that adolescent males have insufficient knowledge about STIs and often struggle to name them or understand the risks to which they are exposed during unsafe and irresponsible sexual practices.

A study conducted in Brazil evaluated young people’s knowledge, attitudes, and practices regarding STIs/AIDS and viral hepatitis, revealing that young people exhibit deficient knowledge about certain STIs as well as the risks of exposure to sex without condoms, particularly in terms of knowledge and practice dimensions. These factors may contribute to increased health vulnerability among male adolescents to STI transmission^(18)^.

Another aspect of the adolescents’ knowledge gap highlighted in this study pertains to the use of the morning-after pill, which was mistakenly regarded as a prophylactic measure against STIs. Consequently, it is concluded that not all information acquired by the adolescents in this study is accurate enough to ensure adequate knowledge about safe sex.

A systematic review supports the findings of this study by elucidating that a large portion of studies report that knowledge about emergency contraception is typically limited to awareness of its existence, without translating into an understanding of where, how, and under what circumstances it should be used. It was also noted that the main sources of information on emergency contraception methods are friends, schools, and media. This underscores the need for reforms in health services to ensure universal access to sexual and reproductive health and reproductive rights^(19)^.

The lack of knowledge and limitations in information regarding safe sex highlight the need for empowering healthcare professionals to act as health educators for the adolescent population. Therefore, it is understood that adolescents with higher levels of knowledge, combined with continuous educational actions on sexual health, are more likely to convert their knowledge about safe sex into effective protected sexual practices, reducing the risk of STIs and unplanned pregnancies^(6, 20)^.

In addition to including targets related to sexual and reproductive health, SDG 3 aims to ensure healthy lives and promote well-being for all at all ages by providing universal access to sexual and reproductive health services with comprehensive sexuality education^(7)^. However, the lack of social protection, combined with weaknesses in safe sex guidance, represents significant obstacles to achieving this goal, resulting in experiences of vulnerability with negative repercussions throughout life.

### Weaknesses in Guidance on Safe Sex Within the Family Context

Although adolescents recognize the importance of safe sex, they also understand the shared responsibility of parents/guardians and their relevance in discussing this topic. Thus, it is evident that family dialogue regarding safe sex remains permeated by taboos and prejudices, resulting in communication barriers that hinder access to knowledge and the ability to make health-promoting sexual decisions.

The family, however, should be considered a safe space responsible for perpetuating ethical and moral values, guiding adolescents as they develop their relationships in and with the world throughout life. This process includes sexual education, with the family playing a role in discussing, guiding, and resolving, whenever possible, the main doubts related to an individual’s sexual development while seeking to identify and avoid the perpetuation of taboos and prejudices that may be harmful to the health and well-being of male adolescents^(21)^.

It is apparent that male adolescents in this study indicated that when dialogue with parents occurs, it is often limited to a focus on condom use for preventing adolescent pregnancy and STI transmission, without addressing their broader concerns and expectations regarding safe sex.

It is crucial to approach comprehensive sexuality education not merely through the lens of disease prevention and unwanted pregnancies but from a perspective that identifies this subject as an integral part of interpersonal and family relationships, capable of creating welcoming and less prejudiced spaces for dialogue^(21)^. This approach seeks to break away from the reproduction of controlling and authoritarian parental attitudes that may have negative consequences on male adolescents’ lives^(22)^.

Therefore, it is necessary to reconsider the importance of family relationships for optimizing strategies in comprehensive sexuality education. The family plays a fundamental role in building solid knowledge for adolescents’ safe sexual practices; however, this topic still represents a challenge to overcome, even today, due to sociocultural contexts ranging from a lack of understanding of the topic to parents’ lack of preparedness for effectively educating their children on sexual matters^(23)^.

Adolescents reported turning to their mothers to discuss safe sex because they found them more approachable for offering guidance. A study conducted in Iran found that not talking with mothers negatively influenced adolescents’ attitudes, noting that most participants who did not engage in dialogue about safe sex with their mothers exhibited risky sexual behaviors^(24)^.

The participatory dialogue with the maternal figure is the result of a daily process, stemming from the increased amount of time mothers spend with their children. These characteristics become evident when considering dialogue as more than merely imposing ideas within a hierarchical relationship; instead, it involves demystifying such situations through interrelated conversations with clear, accessible, and objective dialogues that are embedded within the adolescents’ cultural, social, and religious realities. This approach is capable of discussing, guiding, and addressing possible doubts, taboos, and fears present during this stage of life^(22)^.

Given the scenario faced by male adolescents and their families, it becomes an urgent challenge to achieve the goals established by SDG 3, which focuses on promoting sexual health and well-being through access to information on sexuality. Empowerment through educational health strategies is necessary to create spaces that guarantee the exercise of citizenship and promote self-management in sexual and reproductive health, thereby fostering critical thinking. It is essential for families to receive adequate support and educational resources aimed at recognizing the importance of dialogue for open and inclusive communication, considering adolescents as social beings and protagonists of their life choices^(21)^.

### School as a Privileged Space for Teaching and Learning About Safe Sex for Male Adolescents

As evidenced by the results, adolescents recognize school as a safe and welcoming space for addressing topics related to sexuality, given that the school environment fulfills an educational function that extends beyond curricular knowledge, integrating issues of citizenship and the sociocultural and emotional development of school-age adolescents.

Often, discussions about sexuality are limited to biological aspects, while behavioral, psychological, and motivational components require specific preparation. This aligns with adolescents’ desire for an intersectoral approach within the school context, involving health professionals to provide guidance on the topic. Such findings underscore the importance of an interdisciplinary approach, as sexuality is inherent to adolescent development and must be considered, protected, and respected in all its nuances^(25)^.

Among the few adolescents who reported having access to educational activities, it was noted that the topics covered often focused on condom use and/or STI prevention in a disconnected manner. Literature highlights that many educational programs fail to address specific issues affecting the sexual health of male adolescents, resulting in a disconnect between the presented content and the real experiences of this age group, leading to superficial and disengaged learning^(26)^.

It is crucial to approach comprehensive sexuality education as a transversal theme with a systematic, continuous, and inclusive character. The National Curriculum Parameters (PCN) suggest that sexuality content should be addressed starting from the 5th grade, considering that at this age there is a better understanding of issues related to human sexuality. This approach takes into account curiosity and encourages critical and dialogical thinking among school-aged adolescents, recognizing this topic as a social urgency^(27)^.

Thus, it is essential to foster joint dialogue involving parents/guardians, educators, and health professionals to establish welcoming and informative spaces that address existing doubts in the educational process related to sexuality. This collective effort aims to build a solid social support network that expands existing knowledge and promotes comprehensive health for male adolescents^(6)^.

Another significant challenge faced by male adolescents, when lacking adequate support on the topic within the family and/or school context, is the easy access to erotic content in the virtual environment, which can provide harmful information to their development. Adolescents often rely on information acquired from unreliable sources, such as friends or social media, as a reference, which can perpetuate myths, taboos, and misconceptions about safe and responsible sex^(28)^.

To overcome these challenges, it is crucial to adopt inclusive and gender-sensitive approaches for participatory teaching about safe sex. This involves recognizing and challenging norms of masculinity that can negatively impact the behavior of male adolescents, promoting a healthier and more positive definition of masculinity. Additionally, integrating technology and social media can be an effective way to engage adolescents and involve them in discussions about sexual health in a playful and relevant manner that resonates with their social context^(29)^.

The correlation between teaching and learning about safe sex for male adolescents and SDG 3 is noteworthy, as it aims to ensure healthy lives and promote well-being for all at all ages. By promoting comprehensive, inclusive, and engaging sexuality education within the school setting, nurses contribute to the prevention of STIs, unwanted pregnancies, and other sexual health issues affecting adolescents. This is fundamental for ensuring universal access to sexual and reproductive health services. Moreover, by addressing gender-related stigmas around masculinity, nurses help break down barriers that prevent male adolescents from seeking healthcare and information on sexuality and safe sex, thereby contributing to the achievement of SDG 3 goals^(7)^.

Considering SDG 4, which envisions inclusive, equitable, and quality education for all and promotes lifelong learning opportunities, quality sexual education is vital. Primary care nurses, in alignment with the School Health Program, enable male adolescents to strengthen their prior knowledge and develop skills necessary for social change within their sexual lives. This contributes to building a healthier and more equitable society where everyone has access to the education and information needed to make responsible decisions about their sexual health and well-being^(7)^.

### Study limitations

Given the continental size of Brazil and the multicultural complexity involved in adolescent health issues, a limitation of this study is that it was conducted in only one public educational institution. This reflects territorial constraints and the sociocultural specificities related to the sexuality and sexual behavior of the male adolescent school population studied.

### Contributions to the Field of Nursing

This study aims to equip undergraduate students and the professional practice of Primary Health Care nurses to engage in intersectoral and interdisciplinary efforts in developing educational health strategies within the school context, with a focus on sexual and reproductive health.

## CONCLUSIONS

Addressing safe sex is a social responsibility in the formation of citizenship among school-aged adolescents. However, resistance and a lack of skill in addressing the topic persist, as it continues to be cloaked in taboos and prejudices and often focuses solely on biological aspects, without considering the doubts and concerns experienced by this population group. This situation weakens the creation of welcoming and dialogical spaces within the family environment. It is necessary to consider the multiple masculinities that reshape the complexity and depth of understanding the factors contributing to early and unprotected sexual initiation among adolescents and their partners. The study provided insights into the meanings that male adolescents attribute to safe sex, related to the SDG, with the aim of equipping nurses as health educators on safe sex.

This strengthens the leadership role of male adolescents and their families in breaking the inequities that permeate intersectoral and interdisciplinary care in sexual education, aiming to reduce risk behaviors that may compromise the comprehensive development of adolescents. The limitations in knowledge about safe sex and the lack of critical reflection on the harmful repercussions for this group highlight the need to promote the implementation of public health policies with a direct impact on the lives of male adolescents. It is essential for these policies to be articulated to strengthen the support network, including families and the school environment.

Expanding dialogical spaces for comprehensive sexuality education that includes topics to strengthen safe and responsible sexual practices is necessary. Therefore, promoting intervention studies and the development of educational technologies in sexual and reproductive health for adolescents is essential, aligned with the global goals of Health and Well-Being, Quality Education, and Gender Equality established by the 2030 Agenda.
