# Supplementary material for: Adolescent males’ knowledge about safe sex in light of the Sustainable Development Goals
Source: Rev Bras Enferm. 2025 Jun 20;78(Suppl 1):e20240422. doi: 10.1590/0034-7167-2024-0422 (PMC12185113; doi:10.1590/0034-7167-2024-0422)
Supplement: Translation

# Translation: Conhecimentos de adolescentes masculinos sobre sexo seguro à luz dos Objetivos do Desenvolvimento Sustentável

**DOI:** 10.1590/0034-7167-2024-0422pt

**Authors:** Wallacy Jhon Silva Araújo, Danielle Laet Silva Galvão, Dayanne Caroline de Assis Silva, Gabriela Rodrigues Bragagnollo, Eliane Maria Ribeiro de Vasconcelos, Mariana Boulitreau Siqueira Campos Barros, Estela Maria Leite Meirelles Monteiro

**Affiliations:** I Universidade Federal de Pernambuco. Recife, Pernambuco, Brasil; II Universidade de São Paulo. Ribeirão Preto, São Paulo, Brasil

**Keywords:** Adolescente, Sexualidade, Masculinidade, Desenvolvimento Sustentável, Educação em Saúde

## Abstract

**Objetivos:**

compreender o conhecimento de adolescentes masculinos sobre sexo seguro à luz dos Objetivos do Desenvolvimento Sustentável da Agenda 2030.

**Métodos:**

estudo qualitativo com 18 adolescentes masculinos, com idade entre 15 e 19 anos, em uma escola pública do Recife/Pernambuco, realizado entre os meses de junho e dezembro de 2019. Utilizou-se um instrumento semiestruturado para a realização das entrevistas e a análise temática do conteúdo foi fundamentada no referencial proposto por Bardin.

**Resultados:**

emergiram três categorias temáticas: Conhecimento de adolescentes masculinos sobre sexo seguro; Fragilidades nas orientações sobre sexo seguro no contexto familiar; e Escola como espaço privilegiado para o ensino-aprendizagem sobre sexo seguro para adolescentes masculinos.

**Considerações Finais:**

as limitações no conhecimento sobre sexo seguro geram repercussões prejudiciais à saúde, fragilizando o diálogo familiar e escolar, e comprometendo o entendimento dos fatores de risco que contribuem para a iniciação sexual precoce e desprotegida de adolescentes e seus parceiros(as).

## INTRODUÇÃO

A adolescência é permeada por concepções históricas, sociais e culturais, vinculadas às experiências da sexualidade humana^(1)^. Nessa época, ocorrem experimentações de novos comportamentos, que resultam nas primeiras relações amorosas e sexuais, as quais permitem aos adolescentes buscar o fortalecimento de sua identidade individual^(2)^. Sabe-se que as práticas sexuais transcendem os aspectos biológicos e envolvem características psicossocioculturais, marcadas por imposições sociais que moldam comportamentos sexuais masculinos desprovidos de responsabilidades, de valores éticos e morais, concorrendo para situações de vulnerabilidade^(3)^.

Em razão da existência de tabus, preconceitos e deficiência de conhecimentos sobre sexo seguro, na fase de descoberta das práticas sexuais, a carência de informações e a comunicação ineficaz com o público adolescente contribuem para desencadear comportamentos deficitários de autocuidado em saúde sexual^(4)^.

Com o advento do desenvolvimento tecnológico, houve um aumento na disponibilidade de informações de conteúdo sexual no ambiente virtual^(1)^. Contudo, evidencia-se a exposição ao consumo de informações distorcidas sobre a temática e a não disponibilização de cenários seguros para discussões em ambiente virtual, contribuindo para estimular a iniciação sexual precoce (ISP), desprovida de segurança e responsabilidade, principalmente entre os adolescentes masculinos^(3)^.

Oriundo desse quadro deficitário, a prática de sexo inseguro torna-se recorrente, apresentando como principais desdobramentos as Infecções Sexualmente Transmissíveis (IST), o uso inconsistente de contraceptivos e a gravidez indesejada^(5)^. Esses aspectos podem desencadear repercussões negativas na vida dos adolescentes, das famílias e da sociedade, além de aumentar os custos com a saúde em todos os níveis de atenção^(1)^.

Destarte, ao considerar a garantia dos direitos sexuais e reprodutivos da população adolescente masculina, foram pactuadas metas em consonância com a Agenda 2030 para o Desenvolvimento Sustentável, visando à elaboração de políticas públicas e à implementação de ações voltadas para esse público etário, com o intuito de acabar com as epidemias de Aids e IST; oportunizar a garantia de acesso universal aos serviços de assistência à saúde sexual e reprodutiva, que incluem o planejamento familiar e informações para que todos os meninos e meninas obtenham os conhecimentos e habilidades necessários para seu desenvolvimento integral, mediante a oferta de uma educação de qualidade^(6)^.

Neste contexto, emerge a importância do Programa de Saúde Escolar e de ações educativas em saúde intersetoriais e interdisciplinares, articuladas entre educação e saúde, para ampliar e integrar o contexto escolar, familiar e comunitário na abordagem dialógica sobre a temática^(7)^.

## OBJETIVOS

Compreender o conhecimento de adolescentes masculinos sobre sexo seguro à luz dos Objetivos do Desenvolvimento Sustentável (ODS) da Agenda 2030.

## MÉTODOS

### Aspectos éticos

O projeto de pesquisa obteve aprovação do Comitê de Ética em Pesquisa, conforme preconiza a Resolução Nº 466/12 do Conselho Nacional de Saúde, onde os adolescentes menores de dezoito anos assinaram o Termo de Assentimento Livre e Esclarecido, sendo necessária a anuência formal dos pais/responsáveis para a sua participação, por meio da assinatura do Termo de Consentimento Livre e Esclarecido dos pais/responsáveis antes da coleta de dados. O anonimato e o sigilo das informações pessoais foram garantidos por meio da atribuição de codinomes aos participantes.

### Referencial teórico-metodológico

Utilizou-se como referencial teórico-metodológico a proposta da Agenda 2030 para o alcance das metas dos ODS. A escolha desse referencial possibilitou uma análise embasada no alcance de objetivos que fomentam ações interconectadas em prol da saúde e bem-estar, educação de qualidade e igualdade de gênero^(6)^.

### Tipo de estudo

Estudo descritivo com abordagem qualitativa, norteado pela ferramenta *Consolidated Criteria for Reporting Qualitative Studies* (COREQ)^(8)^.

### Procedimentos metodológicos

A população foi composta por adolescentes autodeclarados do gênero masculino, com idade entre 15 e 19 anos e que já haviam iniciado práticas sexuais. Optou-se por essa faixa etária para permitir uma abordagem minuciosa sobre o tema, com escolares em fase de maturescência. Foram excluídos adolescentes com necessidade de ensino especial, conforme diagnóstico médico apresentado à equipe gestora, e os escolares que já participavam de outros estudos sobre a temática.

O recrutamento dos participantes utilizou a amostragem em bola de neve^(9)^, em que o primeiro participante foi indicado pela coordenação pedagógica por atuar como protagonista escolar, dando sequência à rede de novos integrantes do estudo. A delimitação dos participantes utilizou o critério de saturação teórica^(10)^, constatado na 14ª entrevista, quando não surgiram novas informações relevantes para o estudo. Após a constatação da saturação, optou-se por realizar mais 4 entrevistas para confirmar o processo de saturação de respostas. A composição amostral foi de 18 adolescentes e não houve desistências.

### Cenário do estudo

O estudo foi desenvolvido em uma escola pública de ensino básico do estado de Pernambuco. A escola selecionada oferece educação básica do 5° ano fundamental ao 3° ano do ensino médio, contudo, não dispõe de ações integradas ao Programa Saúde na Escola. A escolha do cenário do estudo considerou o relato da diretoria e da equipe pedagógica da escola, que enfatizou a ocorrência de casos de consumo de álcool e drogas, *bullying,* gravidez na adolescência, ISP e IST.

### Coleta e organização dos dados

A condução das entrevistas ocorreu entre os meses de junho e dezembro de 2019, sendo antecedida pela inserção prévia do pesquisador no ambiente escolar. Foi utilizada a técnica de entrevista em profundidade, com apreensão de dados objetivos referentes à caracterização sociodemográfica dos participantes e uma questão subjetiva. A questão norteadora da entrevista foi previamente validada por três especialistas na área da sexualidade, sendo um enfermeiro, um pedagogo e um psicólogo.

Foi realizado um teste piloto com entrevistas de três adolescentes que não compuseram a amostra final. Esse processo subsidiou as modificações necessárias na apresentação do roteiro de entrevista aplicado. O instrumento semiestruturado para coleta foi subdividido em duas partes: a primeira parte destinada à apreensão de dados sociodemográficos e a segunda parte com a questão geradora da entrevista qualitativa^(11)^: “Quais os conhecimentos você considera serem necessários para que os adolescentes que se autodeclaram do gênero masculino se sintam preparados para a prática sexual segura?”.

As entrevistas ocorreram nos turnos da manhã e tarde, conforme agendamento prévio com a equipe pedagógica, para não comprometer as atividades curriculares. Utilizou-se um ambiente reservado, com duração média de 40 minutos. Os relatos foram registrados mediante dupla gravação e transcrição, e submetidos à validação pelos adolescentes, por meio de leitura e anuência das transcrições realizadas, com agendamento via contato telefônico^(12)^.

### Análise dos dados

Como técnica de categorização e tratamento de dados, utilizou-se a análise de conteúdo temática de Bardin, que contempla três fases cronológicas: pré-análise, em que ocorre a leitura flutuante e organização inicial do corpus da pesquisa; exploração do material, para codificação e categorização; e tratamento dos resultados obtidos e interpretação, subsidiada pelo método reflexivo^(13)^.

## RESULTADOS

Participaram 18 adolescentes autodeclarados masculinos com média de idade de 18,3 anos. Em relação à orientação sexual, 13 adolescentes se reconheceram como heterossexuais, quatro como homossexuais e um como transexual; nove se autodeclararam pretos, cinco pardos, três brancos e um referiu não se importar com a autodeclaração étnico-racial. No que concerne aos aspectos religiosos, cinco eram evangélicos, quatro católicos, um candomblecista, e oito adolescentes referiram não ter religião, porém afirmaram acreditar em Deus.

Além disso, verificou-se que oito adolescentes cursavam o 3° ano, quatro o 2° ano e quatro o 1° ano do ensino médio; também havia dois no 9° ano do ensino fundamental. Houve predominância de participantes do estudo que residiam com os pais e irmãos, e a maioria tinha renda familiar de até dois salários-mínimos.

Com base nos relatos dos participantes, emergiram três categorias temáticas interrelacionadas, intituladas: Conhecimento de adolescentes masculinos sobre sexo seguro; Fragilidades nas orientações sobre sexo seguro no contexto familiar; e Escola como espaço privilegiado para o ensino-aprendizagem sobre sexo seguro para adolescentes masculinos.

### Conhecimento de adolescentes masculinos sobre sexo seguro

Foram evidenciados relatos de desconhecimento sobre a prática do sexo seguro, contribuindo para uma situação de vulnerabilidade diante da eclosão da sexualidade e da ausência de percepção dos riscos à saúde do adolescente. Destaca-se a falta de acessibilidade a conhecimentos que atendam às reais necessidades e especificidades socioemocionais e culturais desse grupo populacional.

*Nunca tive nenhuma informação na tentativa de me explicar sobre sexo seguro de forma eficaz. ‘*Apolo’

*Sabia muito pouco, na verdade quase nada. Naquela época, a única coisa que sabia era que para o sexo acontecer deveria haver penetração.* (Himeneu)

*Eu era muito lerdo. Não sabia de nada. Só ficava jogando videogame. Só depois da minha primeira prática sexual que descobrir que se ejacular-mos dentro da vagina da garota, ocorre o risco de gravidez não planejada.* (Anteros)

*Eu não sabia de nada. Na verdade, penso que não precisamos de conhecimento algum. Não é necessário pesquisar em livros ou internet.* (Afrodite)

Alguns relatos limitaram-se à recomendação de utilização do preservativo como método preventivo. Entretanto, os participantes ainda apresentaram conhecimentos fragilizados sobre a utilização desse método como uma barreira potencial para a prevenção de contaminações relacionadas às 1ST. O frágil conhecimento sobre as 1ST por parte dos adolescentes limita a compreensão dos riscos existentes nas práticas sexuais desprovidas de segurança e enfraquece a adesão ao uso do preservativo.

*Didaticamente eu não tinha nenhum conhecimento, não tinha muita informação do que fazer. A única informação que tinha mesmo era sobre a utilização do preservativo para prevenção de IST, apesar de não as conhecer à fundo.* (Zeus)

*Não tinha noção de praticamente nada. Apenas sabia o que era IST, como por exemplo o HIV, e o que eu deveria usar na hora para prevenção dessa contaminação*. (Éos)

*Não tinha conhecimento. Apenas me falaram sobre o uso do preservativo para prevenção de IST, tais como a própria Aids.* (Himeros)

No que concerne à pílula do dia seguinte, ela foi relacionada como método de contracepção de emergência e, erroneamente, como prevenção de 1ST. Os adolescentes relataram que esse recurso deve ser utilizado na tentativa de corrigir comportamentos de risco que ocorreram ocasionalmente durante relações sexuais sem proteção, que podem levar ao surgimento de gravidez indesejada na adolescência.

Essa concepção fragilizada sobre o uso da pílula do dia seguinte expõe o grupo de adolescentes masculinos a situações de vulnerabilidade relacionadas à saúde sexual e reprodutiva, com repercussões sociais e emocionais, diante do sentimento de preocupação quanto ao risco de uma gravidez não planejada e de contrair IST.

*Se ocorresse de transar sem preservativo, no outro dia a garota teria que tomar a pílula do dia seguinte para evitar gravidez não planejada e contaminação por IST. Eu sabia que se engravidasse a menina eu poderia me prejudicar.* (Hermes)

*Todos nós já sabemos o que acontece durante o sexo e não podemos esquecer jamais de tomar alguns medicamentos no dia seguinte.* (Afrodite)

*O conhecimento que eu tinha era sobre o uso do preservativo e se o mesmo estourasse, deveríamos comprar a pílula do dia seguinte para evitar a contaminação de IST e gravidez não planejada.* (Ares)

### Fragilidades nas orientações sobre sexo seguro no contexto familiar

Embora pareça ser consensual que o ambiente familiar seja um espaço emocional privilegiado para desenvolver e adquirir conhecimentos sobre sexo seguro, a maioria dos participantes refere não dialogar com os pais sobre questões que envolvem a sexualidade. Isso evidencia a perpetuação da fragilidade nas relações familiares, associada aos tabus e preconceitos que circundam a abordagem da sexualidade, dificultando o entendimento do sexo seguro como uma prática saudável vinculada às questões de saúde humana. Contudo, muitos pais e responsáveis são resistentes à abordagem do tema, por considerarem que isso pode estimular a precocidade no ato sexual. Além disso, é possível perceber que os adolescentes se sentem mais confortáveis para dialogar sobre sexo seguro com as mães, devido à relação amorosa que elas mantêm com os filhos, geralmente desprovida de intolerância e preconceito.

*Esses assuntos não eram muito falados, era algo privado aos adultos. Meu pai não gostava muito de conversar comigo sobre esses temas e eu sempre fui tímido para discuti-los, na verdade eu preferia pesquisar na internet sozinho.* (Zeus)

*Meu pai e eu, nunca tivemos tanta intimidade para conversar sobre esse tipo de assunto. Porém, minha mãe sempre abria meus olhos relatando a importância da utilização do preservativo.* (Apolo)

*Meus pais não me disseram que se um dia eu tivesse relação sexual, deveria fazer uso do preservativo para não pegar alguma IST. Devemos conversar com nossos pais sobre esses assuntos, para que os pais possam aconselhar seus filhos, identificando se o mesmo está preparado para iniciar suas atividades sexuais. E acima de tudo, abordar temas como a prevenção de IST.* (Deméter)

### Escola como espaço privilegiado para o ensino-aprendizagem sobre sexo seguro para adolescentes masculinos

Os adolescentes acreditam que a escola seja um ambiente seguro e propício para a aquisição de informações e o desenvolvimento de conhecimentos confiáveis sobre questões de sexualidade; entretanto, evidenciou-se que o acesso a esses conhecimentos ocorre em momentos posteriores à iniciação sexual dos mesmos. Emergiu ainda, entre os adolescentes, o desejo de expressar suas opiniões e pensamentos de forma democrática em relação à utilização do preservativo e à prevenção das IST.

*O adolescente também tem voz e tem o que falar. Vejo que temáticas como essa devam ser trabalhadas no ambiente escolar em forma de rodas de conversa. Eu gostaria que os profissionais médicos ou sexólogos abordassem a temática de IST no ambiente escolar.* (Zeus)

*Nunca tive informações em casa. Eu as adquiri na escola, dois anos após a minha iniciação sexual. Aprendi informações sobre preservativo e os cuidados que são necessários ter durante a colocação do mesmo.* (Pothos)

*Eu tinha muita informação. Aprendi na escola através dos meus professores. Eles sempre me ensinavam sobre a importância de fazer uso do preservativo para me prevenir das IST existentes no mundo.* (Poseidon)

## DISCUSSÃO

Os participantes deste estudo foram caracterizados como adolescentes autodeclarados masculinos, com baixo nível socioeconômico, em sua maioria pertencentes à população preta e com ausência de prática religiosa, o que contribui para a vulnerabilidade social. O estudo evidenciou que esse perfil apresenta uma forte relação com o contexto socio-histórico, que propicia a fragilidade de conhecimentos sobre sexo seguro e práticas sexuais precoces, culminando na ocorrência de situações de vulnerabilidade à saúde da população adolescente^(14)^.

Ao abordar a temática do sexo seguro no contexto masculino, é necessário contemplar os indivíduos pertencentes ao universo das múltiplas masculinidades. Para tanto, é importante considerar que o surgimento da identidade masculina está atrelado à centralidade da ideia de combinação de pluralidade e hierarquia das masculinidades, desconstruindo a concepção de um único padrão de poder. Masculinidades, no plural, são socialmente construídas e variam em diferentes contextos socioculturais ao longo de um determinado período de tempo, em lugares potenciais de identidade e ao longo da vida de qualquer homem^(15)^.

Portanto, a representação de masculinidade pode ser delineada de duas formas: uma tradicional, relacionada às expressões de força e virilidade, enquanto a segunda compreende uma manifestação de aspectos de emocionalidade, sensibilidade, criatividade e imaginação, características frequentemente negadas à identidade masculina em sua dimensão integral e plural^(51)^.

Cabe considerar que as discussões envolvendo igualdade de gênero, conforme evidenciado no ODS 5, têm sido abordadas no universo científico, destacando os direitos femininos frente às posturas machistas construídas socialmente, que contribuem para situações de vulnerabilidade. Para tanto, é fundamental considerar a importância do empoderamento masculino na perspectiva do autoconhecimento, visando estabelecer relações respeitosas, com responsabilidades compartilhadas, propiciando apoio mútuo na construção de relações interpessoais e sexuais seguras com o(a) parceiro(a)^(7)^.

As situações de vulnerabilidade experienciadas pelos adolescentes masculinos em seu contexto social muitas vezes são definidas pelas expectativas culturais tradicionais da masculinidade hegemônica, evidenciando a real necessidade de investimentos de apoio à promoção de conhecimentos adequados e satisfatórios sobre sexo seguro, buscando um desenvolvimento sexual saudável, com vistas a otimizar a educação sexual em sua completude e a derrubar barreiras de acesso aos serviços de saúde sexual e reprodutiva^(16)^.

### Conhecimento de adolescentes masculinos sobre sexo seguro

Os adolescentes do estudo possuem um nível fragilizado de entendimento sobre sexo seguro. Foi observada uma correlação entre o termo sexo seguro e a utilização do preservativo masculino como método de proteção contra a contaminação por 1ST e gravidez indesejada, convergindo com achados de estudos nacionais e internacionais^(6, 17)^. Entretanto, foi evidenciado que eles não se previnem em todas as relações sexuais, demonstrando que os conhecimentos sobre os métodos contraceptivos nem sempre se traduzem em relações sexuais seguras^(17)^.

Ainda neste estudo, observou-se que os adolescentes masculinos possuem conhecimentos insuficientes sobre as 1ST e apresentaram dificuldade em nomeá-las, bem como em compreender os riscos a que estão expostos durante as práticas sexuais desprovidas de segurança e responsabilidade.

Um estudo realizado no Brasil avaliou o conhecimento, a atitude e a prática de jovens em relação às IST/Aids e hepatites virais, constatando que os jovens apresentam conhecimentos deficitários sobre determinadas IST, assim como sobre os riscos de exposição ao sexo sem uso de preservativo, principalmente nas dimensões de conhecimento e prática. Esses fatores podem contribuir para o aumento da vulnerabilidade em saúde dos adolescentes masculinos à contaminação por IST^(18)^.

Outro aspecto presente na defasagem do conhecimento dos adolescentes do estudo refere-se à utilização da pílula do dia seguinte, erroneamente reconhecida como profilática no combate às IST. Dessa forma, conclui-se que nem toda informação adquirida pelos adolescentes do estudo é considerada verídica a ponto de assegurar um conhecimento adequado sobre sexo seguro.

Uma revisão sistemática corrobora os achados deste estudo ao elucidar que grande parte dos estudos relatam que o conhecimento sobre a anticoncepção de emergência está normalmente associado apenas ao fato de saber de sua existência, sem que isso se traduza em saber onde, como e em quais situações utilizá-la. Associado a isso, evidenciou-se que as principais fontes de informação sobre os métodos de anticoncepção de emergência são os amigos, a escola e os meios de comunicação. Dessa forma, percebe-se a necessidade de reformulações nos serviços de saúde, a fim de assegurar o acesso universal à saúde sexual e reprodutiva e aos direitos reprodutivos^(19)^.

O desconhecimento e as limitações de informações sobre sexo seguro denunciam a necessidade de empoderamento dos profissionais de saúde para atuarem como educadores em saúde junto à população adolescente. Destarte, entende-se que adolescentes com níveis mais elevados de conhecimento, associados ao acompanhamento de ações educativas contínuas sobre saúde sexual, têm maior probabilidade de converter o conhecimento sobre sexo seguro em práticas sexuais protegidas efetivas, reduzindo o risco de IST e de gravidez não planejadas^(6, 20)^.

Além de incluir metas relacionadas à saúde sexual e reprodutiva, o ODS 3 visa assegurar uma vida saudável e promover o bem-estar para todos, em todas as idades, oportunizando o acesso universal aos serviços de saúde sexual e reprodutiva com educação abrangente sobre sexualidade^(7)^. Contudo, a falta de proteção social, associada às fragilidades nas orientações sobre sexo seguro, representa obstáculos significativos para alcançar essa meta, culminando na vivência de situações de vulnerabilidade com repercussões negativas ao longo da vida.

### Fragilidades nas orientações sobre sexo seguro no contexto familiar

Apesar de os adolescentes reconhecerem a importância do sexo seguro, eles também compreendem a corresponsabilida-de dos pais/responsáveis e sua relevância na discussão sobre a temática. Dessa forma, nota-se que o diálogo familiar a respeito do sexo seguro ainda é permeado por tabus e preconceitos, culminando em barreiras comunicativas que dificultam o acesso a conhecimentos e discernimento para a tomada de decisões promotoras de saúde sexual.

No entanto, a família deveria ser considerada um espaço seguro e responsável pela perpetuação de valores éticos e morais, capaz de guiar o adolescente para desenvolver suas relações no e com o mundo ao longo da vida. Esse processo inclui a educação sexual, cabendo à família discutir, orientar e dirimir, sempre que possível, as principais dúvidas relacionadas ao desenvolvimento sexual do indivíduo, buscando identificar e evitar a perpetuação de tabus e preconceitos que podem ser prejudiciais à saúde e ao bem-estar dos adolescentes masculinos^(21)^.

Percebe-se que os adolescentes masculinos do estudo evidenciam que, quando ocorre diálogo com os pais, este se limita ao enfoque no uso do preservativo para a prevenção da gravidez na adolescência e da contaminação por IST, desconsiderando as inquietações e expectativas dos adolescentes em relação ao sexo seguro.

É de suma importância tratar a educação integral em sexualidade não apenas sob a ótica da prevenção de doenças e gestações indesejadas, mas também sob uma perspectiva que identifique a temática como parte integrante das relações interpessoais e familiares, capaz de construir espaços de escuta acolhedores e menos preconceituosos^(21)^, rompendo com a reprodução de posturas controladoras e autoritárias dos pais, que podem gerar consequências negativas na vida do adolescente masculino^(22)^.

Portanto, é necessário repensar a importância das relações familiares para a otimização das estratégias de educação integral em sexualidade. A família exerce um papel fundamental na construção de conhecimentos sólidos para práticas sexuais seguras dos adolescentes; contudo, essa temática ainda se configura, mesmo nos dias atuais, como um desafio a ser superado, considerando os contextos socioculturais que decorrem desde a falta de compreensão sobre a temática até o despreparo dos pais para a educação sexual efetiva de seus filhos^(23)^.

Os adolescentes relataram se dirigir às mães para falar sobre sexo seguro, por considerá-las mais acessíveis para oferecer orientações. Um estudo realizado no Irã verificou que não conversar com a mãe influencia negativamente a atitude dos adolescentes, constatando que a maioria dos participantes que não dialogavam sobre sexo seguro com a genitora apresentavam comportamentos sexuais de risco^(24)^.

O diálogo participativo com a figura materna é fruto de uma construção diária, derivada do maior tempo que as mães despendem junto aos seus filhos. Essas características tornam-se evidentes ao considerar o diálogo como algo além do ato de impor ideias em uma relação verticalizada, desmistificando tais situações com conversas inter-relacionadas, por meio de diálogos claros, acessíveis e objetivos, inseridos na realidade cultural, social e religiosa dos adolescentes. Esse tipo de abordagem é capaz de discutir, orientar e sanar possíveis dúvidas, tabus e medos presentes nessa fase da vida^(22)^.

Diante do cenário vivenciado pelos adolescentes masculinos e suas famílias, torna-se um desafio urgente alcançar as metas estabelecidas pelo ODS 3, que versa sobre a promoção da saúde sexual e do bem-estar, mediante o acesso às informações sobre sexualidade. O empoderamento por meio de estratégias educativas em saúde é necessário para criar espaços que garantam o exercício da cidadania e promovam a autogestão em saúde sexual e reprodutiva, oportunizando a formação de pensamentos críticos. É essencial que as famílias recebam apoio e recursos educacionais adequados, com vistas a reconhecer a importância do diálogo para uma comunicação aberta e inclusiva, considerando os adolescentes como seres sociais e protagonistas de suas escolhas de vida^(21)^.

### Escola como espaço privilegiado para o ensino-aprendizagem sobre sexo seguro para adolescentes masculinos

Como evidenciado nos resultados, os adolescentes reconhecem a escola como um espaço seguro e acolhedor para abordar temáticas relacionadas à sexualidade, visto que o ambiente escolar cumpre uma função educacional que integra, além dos conhecimentos curriculares, questões de cidadania e o desenvolvimento sociocultural e emocional dos adolescentes escolares.

Muitas vezes, a temática da sexualidade aborda apenas aspectos biológicos, enquanto os aspectos comportamentais, psicológicos e motivacionais requerem uma preparação específica. Essa situação corrobora com o desejo dos adolescentes de uma atuação intersetorial no contexto escolar, com a participação de profissionais da saúde para orientações sobre a temática. Tal fato evidencia a importância de uma atuação interdisciplinar, visto que a sexualidade é inerente ao desenvolvimento dos adolescentes e precisa ser considerada, protegida e respeitada em todas as suas nuances^(25)^.

Entre os poucos adolescentes que afirmaram ter acesso a atividades educativas, destacou-se que as temáticas abordadas incluem o uso do preservativo e/ou prevenção das IST de forma descontextualizada. Nesse sentido, é destacado na literatura que muitos programas educacionais não abordam questões específicas que afetam a saúde sexual dos adolescentes masculinos, resultando em uma desconexão entre o conteúdo apresentado e as experiências reais desse público etário, levando a uma aprendizagem superficial e descompromissada^(26)^.

É crucial contemplar a educação integral em sexualidade enquanto tema transversal, com um caráter sistemático, contínuo e abrangente. Sendo assim, os Parâmetros Curriculares Nacionais (PCN) orientam que os conteúdos sobre sexualidade devem ser trabalhados a partir do 5° ano, considerando que, a partir dessa faixa etária, ocorre uma melhor compreensão das questões inerentes à sexualidade humana, levando em conta a curiosidade e estimulando a capacidade crítica e dialógica dos adolescentes escolares, ao considerar essa temática uma urgência social^(27)^.

Ressalta-se, então, a importância de uma interlocução conjunta com a participação dos pais/responsáveis, educadores e profissionais da saúde, buscando estabelecer relações dialógicas acolhedoras e esclarecer as dúvidas existentes no processo educativo em sexualidade, com vistas a fomentar uma rede de apoio social sólida que amplie os conhecimentos já existentes e promova uma saúde plena para o adolescente masculino^(6)^.

Outro desafio significativo vivenciado pelos adolescentes masculinos, quando não dispõem de suporte adequado sobre a temática no contexto familiar e/ou escolar, é a facilidade de acesso a conteúdos eróticos no ambiente virtual, que podem fornecer informações prejudiciais ao seu desenvolvimento. Os adolescentes costumam considerar as informações erroneamente adquiridas em fontes não confiáveis, como amigos ou mídias sociais, como referência, o que pode perpetuar mitos, tabus e equívocos sobre sexo seguro e responsável^(28)^.

Para superar esses desafios, é crucial adotar abordagens inclusivas e sensíveis ao gênero masculino para o ensino participativo sobre sexo seguro. Isso envolve reconhecer e desafiar as normas de masculinidade que podem impactar negativamente o comportamento dos adolescentes masculinos, promovendo uma definição mais saudável e positiva de masculinidade. Além disso, a integração de tecnologia e mídias sociais pode ser uma maneira eficaz de instigar os adolescentes e envolvê-los em discussões sobre saúde sexual de uma forma lúdica e relevante para o seu contexto social^(29)^.

Destaca-se a correlação do ensino-aprendizagem sobre sexo seguro para adolescentes masculinos com os ODS 3, que visa garantir uma vida saudável e promover o bem-estar para todos, em todas as idades. Ao promover uma educação integral em sexualidade de maneira lúdica, inclusiva e abrangente no âmbito escolar, o enfermeiro contribui para a prevenção de IST, gravidezes indesejadas e outros problemas de saúde sexual que envolvem os adolescentes. Isso é de fundamental importância para garantir o acesso universal aos serviços de saúde sexual e reprodutiva. Além disso, ao abordar estigmas de gênero relacionados à masculinidade, o enfermeiro mobiliza a superação de barreiras que impedem os adolescentes masculinos de buscar cuidados de saúde e informações sobre sexualidade e sexo seguro, contribuindo assim para o alcance das metas do ODS 3^(7)^.

Ao considerar o ODS 4, vislumbra-se a garantia de uma educação inclusiva, equitativa e de qualidade para todos, promovendo oportunidades de aprendizagem ao longo da vida. Ao promover uma educação sexual de qualidade, o enfermeiro da atenção primária, em consonância com o Programa Saúde na Escola, possibilita que os adolescentes masculinos fortaleçam seus conhecimentos prévios e desenvolvam habilidades necessárias para a mudança social em seu contexto de vida sexual. Isso contribui para a construção de uma sociedade mais saudável e igualitária, onde todos têm acesso à educação e informações necessárias para a tomada de decisões responsáveis sobre sua saúde sexual e bem-estar^(7)^.

### Limitação do estudo

Ao reconhecer a dimensão continental do território brasileiro e a complexidade multicultural que envolve as questões de saúde da população adolescente, emerge como limitação do estudo o fato de ter sido desenvolvido em apenas uma instituição de ensino público, retratando as delimitações territoriais e as especificidades socioculturais que envolvem as questões de sexualidade e comportamento sexual dos adolescentes escolares masculinos participantes do estudo.

### Contribuições para a área da enfermagem

Instrumentalizar a formação de graduandos e a prática profissional de enfermeiros da Atenção Primária em Saúde para uma atuação intersetorial e interdisciplinar no desenvolvimento de estratégias educativas em saúde no contexto escolar, com ênfase em saúde sexual e reprodutiva.

## CONSIDERAÇÕES FINAIS

A abordagem sobre sexo seguro constitui uma responsabilidade social na formação da cidadania de adolescentes escolares. Entretanto, ainda foram evidenciadas resistências e inabilidades em tratar a temática, que permanece revestida de tabus e preconceitos e centralizada nos aspectos biológicos, desconsiderando as dúvidas e inquietações vivenciadas por esse grupo populacional. Tal situação fragiliza a construção de espaços acolhedores e dialógicos no ambiente familiar. É necessário considerar as múltiplas masculinidades que redimensionam a complexidade e profundidade no entendimento dos fatores que contribuem para a ISP e desprotegida de adolescentes e seus parceiros(as).

O estudo proporcionou a aquisição de informações sobre os significados que os adolescentes masculinos atribuem ao sexo seguro, associados aos ODS, com vistas a instrumentalizar o enfermeiro como educador em saúde sobre sexo seguro, de modo a fortalecer o protagonismo dos adolescentes masculinos e suas famílias para romper as iniquidades que permeiam os cuidados intersetoriais e interdisciplinares em educação sexual, visando à diminuição de comportamentos de risco que podem comprometer o desenvolvimento integral do adolescente.

As limitações nos conhecimentos sobre sexo seguro e a carência de reflexão crítica sobre as repercussões prejudiciais para este grupo evidenciam a necessidade de fomentar a implementação de políticas públicas de saúde com impacto direto na vida dos adolescentes masculinos, ressaltando a importância de serem articuladas para fortalecer a rede de apoio, como a família e o ambiente escolar.

É necessário favorecer a ampliação de espaços dialógicos para uma educação integral em sexualidade, que inclua tópicos que fortaleçam as práticas sexuais seguras e responsáveis. Portanto, é imprescindível o fomento de estudos de intervenções e o desenvolvimento de tecnologias educacionais em saúde sexual e reprodutiva com adolescentes, em consonância com as metas globais de Saúde e Bem-Estar, Educação de Qualidade e Igualdade de Gênero, pactuadas na Agenda 2030.
